# Clinical efficacy of neoadjuvant chemohormonal therapy combined with laparoscopic radical prostatectomy in high-risk Prostate Cancer

**DOI:** 10.12669/pjms.38.8.5469

**Published:** 2022

**Authors:** Peng Zhang, Shengyong Cai, Chengquan Yan, Lin Li

**Affiliations:** 1 Peng Zhang, Department of Urology, Tangshan Gongren Hospital, No.27 Wen Hua Road, Tangshan 063003, Hebei, China; 2 Shengyong Cai, Department of Urology, Tangshan Gongren Hospital, No.27 Wen Hua Road, Tangshan 063003, Hebei, China; 3 Chengquan Yan, Department of Urology, Tangshan Gongren Hospital, No.27 Wen Hua Road, Tangshan 063003, Hebei, China; 4 Lin Li, Department of Urology, Tangshan Gongren Hospital, No.27 Wen Hua Road, Tangshan 063003, Hebei, China

**Keywords:** Neoadjuvant chemohormonal therapy, Laparoscopic radical prostatectomy, High-risk prostate cancer

## Abstract

**Objectives:**

To evaluate the clinical efficacy of neoadjuvant chemohormonal therapy (NCHT) combined with laparoscopic radical prostatectomy in high-risk prostate cancer (PCa).

**Methods:**

A randomized controlled trial was used in this study. Eighty patients with high-risk PCa treated in Tangshan Gongren Hospital from January 2017 to January 2019 were selected and randomly divided into two groups. The control group was given neoadjuvant endocrine therapy, while the research group was added NCHT to the control group. Three months later, the patients of two groups underwent laparoscopic radical prostatectomy. The changes of surgical indicators, adverse drug reactions, incidence of lower urinary tract symptoms, biochemical recurrence rate after follow-up, PSA progression-free survival and incidence of surgical complications were compared between the two groups.

**Results:**

After NCHT, the PSA level and prostate volume in the research group decreased significantly than those in the control group (*P* = 0.00). Surgical duration, postoperative hospital stay and retention time of drainage tube were significantly shorter and intraoperative blood loss was significantly less in the research group than those in the control group (*P* = 0.00). The incidence of lower urinary tract symptoms, biochemical recurrence and surgical complications in the research group were significantly lower than those in the control group, and the early recovery rate of urinary control and progression-free survival were significantly better than those in the control group (*P* < 0.05).

**Conclusion::**

NCHT combined with laparoscopic radical prostatectomy is a safe and effective treatment for high-risk PCa, which is worthy of promotion in clinical practice.

## INTRODUCTION

Prostate cancer (PCa) is one of the most common malignant tumors of the urinary system, with the highest incidence in all male malignant tumors in European and American countries.^1^ In addition, with the aging of the population and the change of living and eating habits, it presents an increasing trend. According to the recurrence rate and the impact on patients, PCa is divided into low-risk, medium-risk and high-risk.^2^ With the continuous progress of treatment methods, the threat of medium and low-risk PCa to the survival of patients is decreasing. High-risk and locally advanced PCa is the main cause thr

Laparoscopic radical prostatectomy is the most effective method to cure localized PCa,^4^ but its therapeutic effect in high-risk PCa is still controversial.^5^ With the in-depth study on the clinical treatment mode of PCa and the rapid development of minimally invasive technology, there are fewer and fewer surgical complications. Increasing evidence shows that the traditional preoperative neoadjuvant endocrine therapy can degrade the tumor, reduce the stage, improve the tumor resection rate and decrease the positive rate of cutting edge, thus providing opportunities of radical surgery for patients with local progression, However, it can not change the overall survival rate of patients.^6^ Therefore, we performed laparoscopic radical prostatectomy after neoadjuvant chemohormonal therapy (NCHT). The results suggest that it has certain advantages over neoadjuvant endocrine therapy alone in patients with high-risk PCa.

Our objective was to evaluate the clinical efficacy of Neoadjuvant chemo hormonal therapy (NCHT) combined with laparoscopic radical prostatectomy in high-risk prostate cancer (PCa).

## METHODS

A randomized controlled trial was used in this study. Eighty patients with high-risk PCa treated in Tangshan Gongren Hospital from January 2017 to January 2019 were selected and randomly divided into two groups, with 40 patients in each group. In the research group, the patients aged 55-78 years (average, 60.74 ± 8.14 years), and the patients in the control group aged 58-80 years (average, 61.08 ± 8.13 years). The general data showed no significant differences between the two groups, suggesting comparability (Table-I).

**Table-I:** Comparison of general data between research group and control group (X̄ ± S) n =40.

Indicator	Research group	Control group	t/χ^2^	*P*
Age (year)	65.15 ± 7.63	68.00 ± 7.54	1.68	0.10
BMI (kg/m^2^)	23.53 ± 2.81	24.07 ± 2.61	0.89	0.38
Initial PSA (ng/ml)	63.08 ± 21.76	58.82 ± 19.03	0.93	0.35
Initial prostate volume (ml)	54.72 ± 13.87	52.13 ± .94	0.86	0.39
Initial Gleason score	7.73 ± 1.06	7.95 ± 1.20	0.87	0.40
***Clinical stage***				
T2 (n,%)	26	23	0.47	0.49
T3 (n,%)	14	17		
Pelvic lymph node metastasis (n,%)	18	21	0.45	0.50

*P* > 0.05.

### Inclusion criteria:

- Patients with PCa diagnosed by the pathological results of prostate puncture;
- Patients with clinical data meeting the diagnostic criteria of high-risk PCa^7^;
- Patients aged < 80 years;
- Patients and their family members with willingness and ability to cooperate with the study, and good treatment compliance;
- Patients with complete clinical and imaging data;
- Patients signing the informed consent

### Exclusion criteria:

- Patients combined with severe underlying diseases and intolerance to surgery;
- Patients combined with malignant tumors in other parts;
- Patients with mental disorders and inability to cooperate with the study;
- Patients with incomplete clinical data;
- Patients previously receiving radiotherapy or chemotherapy
- Patients with a previous history of pelvic surgery;
- Patients allergic to the drugs involved in the study.

### Ethical Approval:

The study was approved by the Institutional Ethics Committee of Tangshan Gongren Hospital on March 10, 2017 (No.[2017]031), and written informed consent was obtained from all participants.

### Treatment Methods:

The control group was firstly given neoadjuvant endocrine therapy after diagnosis. The specific regimen was oral bicalutamide 50 mg/time, once a d, for three consecutive months, and injection of goserelin acetate 10.8 mg, once every three months. Three months later, laparoscopic radical prostatectomy was performed.^8^ Under general anesthesia, a surgical incision was made 2cm above the umbilicus, and the trochar and laparoscopic lens were inserted, with the pneumoperitoneum pressure maintained at 15 mmHg. Other trocars were placed under the guidance of the laparoscopic lens. In the trendelenburg position, the pelvic fascia was cut open, and the prevesical space and bilateral intrapelvic fascia were freed. The dorsal venous complex (DVC) was ligated using an absorbable suture. The bilateral bladder neck was separated at the proximal basal end of the prostate. Both sides of the bladder neck were separated deep to expose the posterior seminal vesicle gland and vas deferens. The anterior wall of the bladder neck was opened to lift the urethra, and the posterior lip of the bladder neck was cut open to expose the posterior vas deferens and seminal vesicle gland. The vas deferens was disconnected on both sides, the posterior wall of the prostate was separated to open the Denonvilliers’ fascia, and the rectal wall was bluntly separated to the tip of the prostate. The apical urethra was freed and disconnected, and the prostate was finally removed. The bladder neck and posterior urethra were anastomosed with absorbable sutures, and the urinary catheter was placed. The external iliac obturator and internal iliac common iliac and presacral lymph nodes in the bilateral pelvic cavity were dissected.

After diagnosis, the research group was additionally treated with NCHT based on the treatment in the control group. The specific regimen was additionally estramustine phosphate capsules, 7-14 mg/kg, oral administration in two or three times, for four consecutive weeks, and docetaxel 30 mg/m^2^, once a week for six consecutive weeks.^9^ Three months later, the patients underwent laparoscopic radical prostatectomy. The surgical method was the same as that of the control group.

### Observation Indicators:

***Comparative analysis of perioperative indicators:*** Preoperative PSA level, preoperative prostate volume, surgical duration, intraoperative blood loss, postoperative hospital stays, retention time of drainage tube and positive rate of cutting edge were observed in the two groups. The differences in perioperative relevant indicators were compared between the two groups.

***Adverse drug reaction assessment:*** The adverse drug reactions after the treatment cycle in the two groups were recorded, including

***The incidence of lower urinary tract symptoms*** such as painful urination, hematuria, frequent urination and urgent urination after postoperative urinary catheter removal were compared and analyzed between the two groups.

***Comparative analysis of follow-up indicators:*** All the patients were followed up for 24-40 months, and the recovery of urinary control, the rate of biochemical recurrence, PSA progression-free survival and the incidence of surgical complications were observed in the two groups. The Clavien classification of surgical complications was used^10^: *Grade-I:* complications needing no drug therapy, surgery, endoscopy, intervention, etc.; *Grade-II:* complications needing drug therapy; *Grade-III:* complications needing surgery, endoscopy or radiotherapy; *Grade-IV:* life-threatening complications (including central nervous system complications) needing treatment in the intensive care unit; *Grade-V:* death.

### Statistical Analysis:

All data were statistically analyzed using SPSS 20.0. The measurement data were expressed as (X̄ ± S). Inter-group data were analyzed using the two independent samples t-test, and intra-group data with the paired t-test. The rates were compared using the χ^2^ test, repeated measurement data were analyzed by the analysis of variance, and pairwise comparison was performed with the LSD-t test. *P* < 0.05 was considered statistically significant.

## RESULTS

After NCHT, the PSA level and prostate volume in the research group decreased significantly than those in the control group (*P* = 0.00). Surgical duration, postoperative hospital stay and retention time of drainage tube were significantly shorter and intraoperative blood loss was significantly less in the research group than those in the control group (*P* = 0.00). The postoperative pathological results showed no significant difference in the positive rate of cutting edge between the two groups (*P* = 0.17) (Table-II).

**Table-II:** Comparative analysis of perioperative indicators between two groups (X̄ ± S) n = 40.

Group	Research group	Control group	t/χ^2^	*p*
Preoperative PSA (ng/ml)*	8.43 ± 3.79	.82 ± 19.03	16.42	0.00
Preoperative prostate volume (ml)*	30.26 ± 10.14	37.13 ± 11.94	8.41	0.00
Surgical duration (min)*	143.55 ± 32.61	187.25 ± 45.08	4.97	0.00
Intraoperative blood loss (ml)*	0.74 ± 24.08	153.96 ± 26.71	5.84	0.00
Postoperative hospital stay (d)*	.30 ± 2.46	15.86 ± 3.39	5.38	0.00
Retention time of drainage tube (d)*	4.72 ± 1.08	6.55 ± 1.24	7.04	0.00
Positive rate of cutting edge (%)	3 (7.5%)	7 (17.5%)	1.83	0.17

* P < 0.05.

After treatment, the incidence of adverse drug reactions was 27.5% in the research group and 15% in the control group, without statistically significant difference (*P* = 0.17).Table-III. The incidence of lower urinary tract symptoms after postoperative urinary catheter removal in the research group was 17.5%, which was significantly lower than 40% in the control group (*P* = 0.02). Table-IV.

**Table-III:** Comparative analysis of adverse drug reactions between two groups after treatment (X̄ ± S) n = 40

Group	WBC reduction	Gastrointestinal reactions	Peripheral neuritis	Liver function injury	Incidence
Research group	3	3	2	3	11 (27.5%)
Control group	0	4	0	2	6 (15%)
χ^2^					1.87
P					0.17

P > 0.05.

**Table-IV:** Comparative analysis of postoperative lower urinary tract symptoms between two groups (X̄ ± S) n = 40

Group	Hematuria	Painful urination	Frequent urination	Urgent urination	Total
Research group	2	2	3	0	7 (17.5%)
Control group	3	7	5	1	16 (40%)
χ^2^					4.94
P					0.02

P < 0.05.

No significant difference was found in follow-up time between the two groups (*P* = 0.13). The rate of biochemical recurrence in the research group was 15%, which was significantly lower than 35% in the control group (*P* = 0.04). The incidence of surgical complications was .5% in the research group and 32.5% in the control group (*P* = 0.03). The early recovery rate of urinary control in the research group was 67.5%, which was significantly higher than 45% in the control group (*P* = 0.04). Progression-free survival in the research group was significantly longer than that in the control group (*P* = 0.00) (Table-V).

**Table-V:** Comparative analysis of follow-up indicators between two groups (X̄ ± S) n = 40.

Group	Research group	Control group	t/χ^2^	*P*
Follow-up time (month)	39.63 ± 7.35	36.67 ± 7.92	1.73	0.13
Biochemical recurrence (n, %) *	6 (15%)	14 (35%)	4.27	0.04
Progression-free survival (month)*	27.58 ± 8.73	21.35 ± 7.62	3.40	0.00
Surgical complications*	5 ( .5%)	13 (32.5%)	4.59	0.03
Grade I (n)	3	7		
Grade II (n)	1	4		
Grade III (n)	0	1		
Early urinary control (n, %)*	27 (67.5%)	18 (45%)	4.11	0.04

* P < 0.05, early urinary control = recovery of urinary control 1 month after surgery.

## DISCUSSION

PCa is the most common malignant tumor of the male genitourinary system, and patients with high-risk PCa account for more than 30% of the total number of newly diagnosed patients.^11^ The 5-year survival rate of patients with high-risk advanced PCa is only 30%. For the treatment of high-risk PCa, there is no recognized single treatment. Radical prostatectomy is the main method to cure PCa. Researchers in the past tended to recommend radical prostatectomy for patients with low-risk localized PCa, and then extended the indications to moderate-risk PCa. Now, studies have begun to carry out comprehensive treatment including radical prostatectomy in patients with locally advanced PCa and even oligometastatic PCa.^13^ Dearnaley et al. and their colleagues believe that^14^ if patients with high-risk PCa only undergo radical surgery, the postoperative biochemical recurrence rate can reach 5%-70%. Comprehensive treatment including surgery, radiotherapy, neoadjuvant therapy and adjuvant therapy is expected to improve the therapeutic effect in patients. Therefore, many researchers have conducted extensive research and exploration on neoadjuvant therapy for PCa.

Neoadjuvant therapy for PCa refers to adjuvant chemotherapy, endocrine therapy and radiotherapy before surgical resection. The results of clinical trials suggest that neoadjuvant therapy before radical surgery can decrease the positive rate of surgical cutting edge, reduce tumor stage, reduce tumor volume, increase the chance of radical resection and decrease postoperative lymph node metastasis.^15^ The mechanism of traditional preoperative neoadjuvant endocrine therapy is to change the survival environment of cancer cells using total androgen blockade, leading to cancer cell apoptosis, which can degrade the tumor, reduce the stage, shrink the tumor volume, improve the tumor resection rate and decrease the positive rate of cutting edge, so as to provide radical surgery opportunities for locally advanced patients.^16^ However, a meta-analysis by Greenberger et al.^17^ demonstrated that preoperative neoadjuvant endocrine therapy could not improve progression-free survival or overall survival of patients. The residual tumor cells will continue to proliferate or resist endocrine therapy, limiting the therapeutic effect. The use of androgen deprivation therapy or brachytherapy has the same therapeutic effect. Neoadjuvant chemotherapy seems to be feasible and safe at the test dose, but the severity of urinary incontinence may be higher than that of radical prostatectomy alone.^18^ Tafuri et al.^19^ do not recommend preoperative neoadjuvant endocrine therapy alone in that it does not provide any survival advantage.

NCHT refers to the treatment method combining endocrine therapy and chemotherapy before radical surgery, the mechanism of which is that endocrine therapy can select and induce the production of androgen-independent cells. Combined with neoadjuvant chemotherapy, these cells can be early inhibited and killed in time, thereby further reducing the load of cancer cells, tumor volume and the pathological grade of the tumor. Reducing the production of androgen-independent cells can obtain better long-term survival benefits.^20^ Docetaxel combined with androgen deprivation therapy has been proved to be an effective treatment method for hormone-sensitive metastatic PCa.^21^ The meta-analysis of Kuderer et al.^22^ showed that the systematic chemotherapy scheme based on docetaxel could reduce the risk of death in patients with advanced PCa. Some studies tried to use this scheme to carry out neoadjuvant chemotherapy for high-risk locally advanced PCa firstly, and then radical prostatectomy. In hormone-sensitive PCa, early intervention of docetaxel-based chemotherapy ultimately benefits the overall survival rate of patients. Estramustine is a combination of estradiol and nitrogen mustard, with estrogen activity weaker than estradiol, but anti-tumor activity higher than other alkylating agents. It mainly exists in the form of oxide isomers estrone and nitrogen mustard in the body. Both forms accumulate in the prostate and play a certain role in PCa.^23^ Compared with neoadjuvant chemotherapy and neoadjuvant endocrine therapy alone, NCHT may reduce the risk of biochemical recurrence in patients with PCa.^24^ In addition, McKay^25^ believes that NCHT has a good pathological response, but it needs longer follow-up for evaluation. Moreover, a large-scale adjusted analysis of the high-risk PCa population by Tosco et al.^26^ demonstrated that preoperative neoadjuvant therapy significantly reduced PCa-related mortality.

Our study confirmed that the PSA level and prostate volume of the patients after NCHT decreased significantly than those in the control group (*P* = 0.00). Surgical duration, postoperative hospital stay and retention time of drainage tube were significantly shorter and intraoperative blood loss was significantly less in the research group than those in the control group (*P* = 0.00). The incidence of adverse drug reactions was 27.5% in the research group and 15% in the control group, without a statistically significant difference (*P* = 0.17). The incidence of lower urinary tract symptoms after urinary catheter removal in the research group was 17.5% and 40% in the control group, with a statistically significant difference (*P* = 0.02). The rate of biochemical recurrence in the research group was 15%, which was significantly lower than 35% in the control group (*P* = 0.04). The incidence of surgical complications was 0.5% in the research group and 32.5% in the control group (*P* = 0.03). The early recovery rate of urinary control in the research group was 67.5%, which was significantly higher than 45% in the control group (*P* = 0.04). Progression-free survival in the research group was significantly longer than that in the control group (*P* = 0.00).

### Limitations to this study:

Firstly, the sample size is small. Secondly, only bicalutamide and goserelin, the most commonly used endocrine therapy drugs in clinic, were included in the study. However, new drugs for endocrine therapy of PCa were not involved. Additionally, PCa is a chronic disease with a long course of disease, but the follow-up time of our study is still short. With increasing the sample size, we will further improve the research content on the combination of new endocrine therapeutic drugs and chemotherapy, and prolong the follow-up time, so as to make a more objective evaluation on the effect of the treatment scheme on the surgery and the long-term effect.

## CONCLUSION

In conclusion, compared with neoadjuvant endocrine therapy combined with laparoscopic radical prostatectomy, NCHT combined with laparoscopic radical prostatectomy in patients with high-risk PCa can decrease preoperative PSA level, reduce prostate volume, shorten surgical duration, reduce intraoperative blood loss, and shorten postoperative extubation time and hospital stay. Additionally, it does not obviously increase the incidence of adverse reactions, reduces the incidence of surgical complications, benefits the early recovery of urinary control, and prolongs progression-free survival. Therefore, it is a safe and effective treatment method.

### Authors’ Contributions:

**PZ &** **LL:** designed this study, prepared this manuscript, are responsible and accountable for the accuracy and integrity of the work.

**SC:** Collected and analyzed clinical data,

**CY:** Data analysis**,** significant contribution to revise this manuscript.

***Please note:*** Significant contribution alone does not qualify one to be listed as an author.
